# Engineering a Bioelectronic Hybrid Heart with Integrated Sensing and Electrical Modulation

**DOI:** 10.1002/advs.78395

**Published:** 2026-10-22

**Authors:** Itai Cabilly, Hadas Oved, Ron Feiner, Nadav Noor, Moran Yadid, Melanie Flury, Assaf Shapira, Tal Dvir

**Affiliations:** ^1^ The Biotechnology Unit, Shmunis School of Biomedicine and Cancer Research, Faculty of Life Sciences Tel Aviv University Tel Aviv Israel; ^2^ The Center for Nanoscience and Nanotechnology Tel Aviv University Tel Aviv Israel; ^3^ Sagol Center for Regenerative Biotechnology Tel Aviv University Tel Aviv Israel; ^4^ Sagol School for Neuroscience Tel Aviv University Tel Aviv Israel; ^5^ School of Biomedical Engineering, Faculty of Engineering Tel Aviv University Tel Aviv Israel

**Keywords:** bioelectronics, biofabrication, cardiac tissue engineering, decellularization, extracellular matrix, induced pluripotent stem cell, nanotechnology, regenerative medicine, stimulation, tissue engineering

## Abstract

Cardiovascular diseases remain the leading cause of mortality worldwide, motivating the development of advanced therapeutic strategies, including engineered cardiac substitutes. Here, we report a bioelectronic hybrid heart that integrates engineered cardiac tissue with embedded electrophysiological sensing and stimulation capabilities. A decellularized cardiac scaffold was cellularized by injection of induced pluripotent stem cell‐derived cardiomyocytes and assembled with a cell‐laden, three‐dimensional‐printed extracellular matrix coating containing a custom‐designed electronic mesh. After 14 days of culture, the embedded electrodes recorded spontaneous cardiac activity, while localized electrical stimulation affected the tissue, enabling pacing at controlled frequencies. Calcium imaging demonstrated spatial activation across the engineered tissue during pacing. This platform establishes a foundation for integrating real‐time monitoring and electronic functional regulation into engineered cardiac constructs and represents an initial step toward bioelectronic organ engineering.

## Graphical abstract

A bioelectronic hybrid heart is engineered by integrating iPSC‐derived cardiomyocytes, a decellularized cardiac scaffold, a cell‐laden ECM coating, and a flexible electronic mesh. The embedded electrodes enable real‐time recording of spontaneous electrical activity and localized stimulation, allowing controlled pacing and modulation of activation patterns. This platform establishes a foundation for electronically monitored and regulated engineered organs.

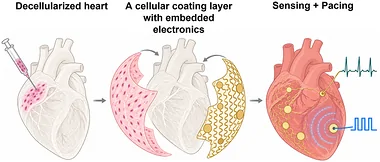

## 1 Introduction

Cardiovascular diseases remain a leading cause of morbidity and mortality worldwide, and for patients with end‐stage heart failure, transplantation is still the definitive treatment option [1]. However, the severe shortage of donor organs continues to drive the search for alternative strategies for heart replacement [2]. Over the past decades, cardiac tissue engineering has progressed substantially, enabling the generation of engineered myocardial patches and other constructs that recapitulate selected structural and functional features of native myocardium [3, 4, 5, 6, 7, 8, 9]. Yet, despite these advances, the fabrication of a whole engineered heart remains one of the central unresolved challenges in regenerative medicine.

Among the available strategies for whole‐heart bioengineering, decellularized native hearts remain particularly attractive because they preserve the hierarchical extracellular matrix architecture, chamber geometry, and vascular tree of the organ [10]. In the seminal work by Ott et al., perfusion decellularization yielded acellular whole‐heart scaffolds that retained native cardiac structure and could subsequently be recellularized to produce constructs showing contraction, drug responsiveness, and early pump function [11]. More recent studies further reinforced the value of decellularized cardiac extracellular matrix as a biologically instructive scaffold and highlighted the broader promise of whole‐heart engineering, even though fully mature organ function has not yet been achieved [12]. Until fully functional engineered hearts can be realized, the integration of sensing and stimulation capabilities may provide a valuable strategy to monitor, regulate, and enhance the performance of these developing cardiac constructs.

Bioelectronics provides an attractive route toward this goal. Previous studies, including work from our group, demonstrated that flexible multifunctional electronics can be integrated with engineered cardiac tissues to enable online monitoring and active modulation of tissue behavior [13]. In particular, the microelectronic cardiac patch demonstrated that embedded free‐standing electronics could record electrical activity, provide on‐demand electrical stimulation within engineered cardiac tissue, and spatiotemporally release essential molecules [14, 15]. Such tissue‐electronics interfaces have emerged as a framework for combining living tissues with embedded enhanced capabilities [16, 17, 18, 19]. However, most of these advances have been demonstrated at the tissue level, whereas translation to a whole‐heart platform introduces additional challenges related to anatomical conformity, three‐dimensional scaffold integration, and compatibility with organ‐scale tissue formation.

Here, we present a bioelectronic whole‐heart construct that integrates iPSC‐derived cardiomyocytes (Figure S1) with a decellularized cardiac scaffold, a custom extracellular matrix (ECM)‐based hydrogel coating, and a flexible, freestanding electronic mesh engineered according to cardiac anatomy and contraction vectors. By embedding electronics directly within a biologically derived whole‐organ environment, this approach provides a strategy to integrate structural, biological, and electronic functions into a single, integrated cardiac system. The resulting hybrid construct is designed not only to support tissue formation and maturation but also to enable real‐time electrophysiological sensing and localized electrical stimulation across anatomically relevant regions of the heart (Figure 1).

**FIGURE 1:**
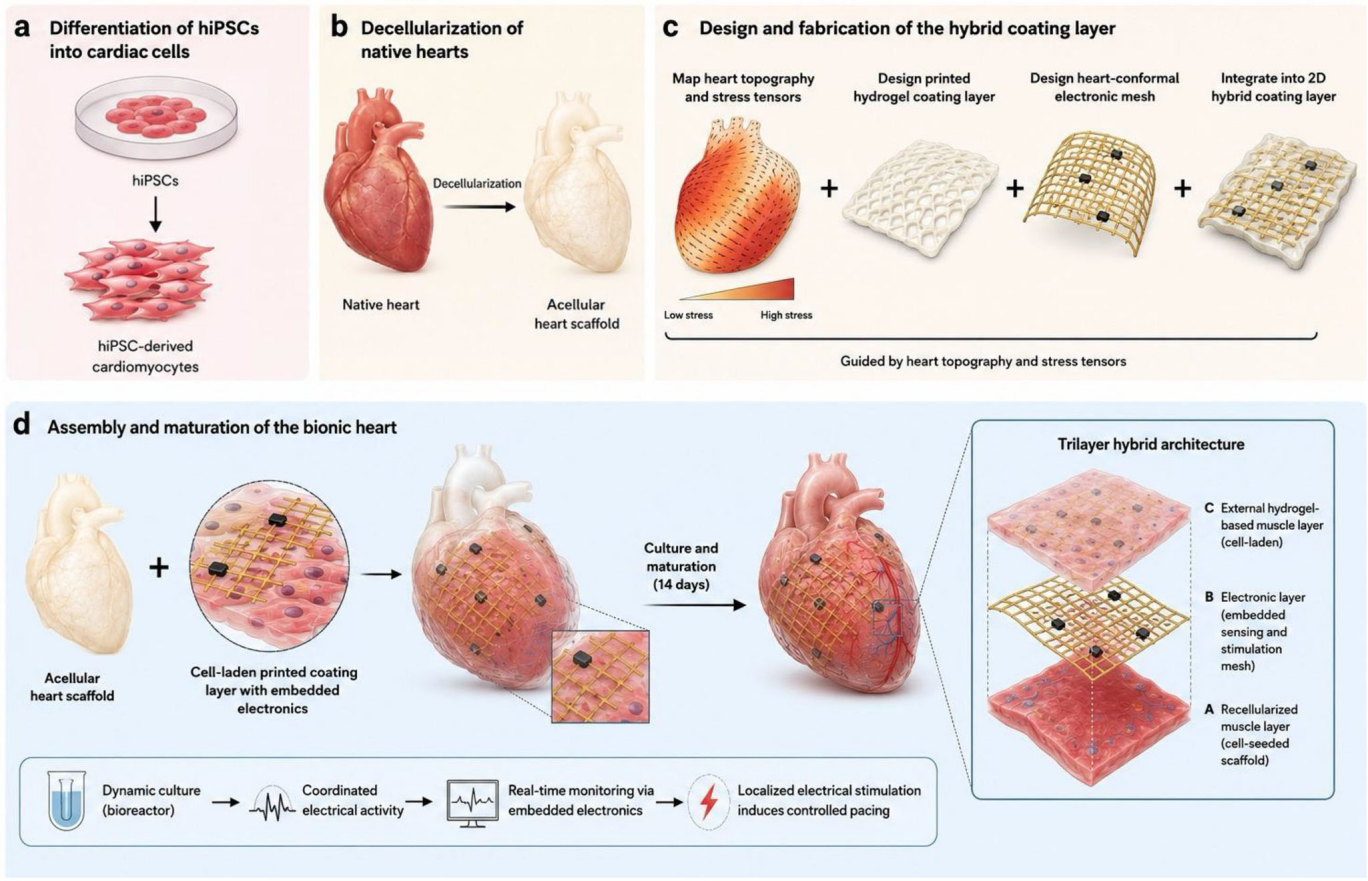
Schematic overview of the bionic heart concept and fabrication strategy. (a) Differentiation of hiPSCs into cardiac cells for construct cellularization. (b) Decellularization of native hearts to generate an acellular whole heart scaffold. (c) Generation of a two‐dimensional map of heart topography and contraction‐related stress tensors, used to guide fabrication of the printed hydrogel coating layer and the heart‐conformal electronic mesh, followed by their integration into a two‐dimensional hybrid coating layer. (d) Assembly of the bionic heart by combining a repopulated decellularized heart with a cell‐laden printed coating layer containing embedded electronics, followed by culture and maturation. The resulting construct is designed as a trilayer hybrid system composed of a recellularized muscle layer, an intermediate electronic layer, and an external hydrogel‐based muscle layer.

### 1.1 Whole‐Heart Decellularization Preserves Native Cardiac Architecture and Vasculature

Whole rat hearts were isolated and decellularized via coronary perfusion to generate acellular scaffolds for bioelectronic heart engineering. The decellularization process progressively removed cellular content while preserving the overall anatomical structure of the organ, ultimately yielding a translucent whole‐heart extracellular matrix scaffold (Figure 2a–c and Figure S2a,b). Perfusion of the decellularized hearts with dye demonstrated preservation of the native coronary vasculature and continuity of the vascular tree throughout the construct (Figure 2d). Macroscopic examination of the whole‐heart scaffold and its sections further confirmed the preservation of chamber‐level morphology and overall cardiac geometry following decellularization (Figure 2e and Figure S2a,b). Scanning electron microscopy revealed that the native extracellular matrix microarchitecture was retained across multiple anatomical regions, including the left ventricle, interventricular septum, right ventricle, and atria, where a porous fibrous ultrastructure characteristic of cardiac ECM remained evident (Figure 2f and Figure S2c). Collectively, these results demonstrate the successful generation of a structurally preserved whole‐heart scaffold that retains both organ‐scale anatomical organization and microscale extracellular matrix features essential for subsequent biohybrid tissue assembly and integration with embedded electronics.

**FIGURE 2:**
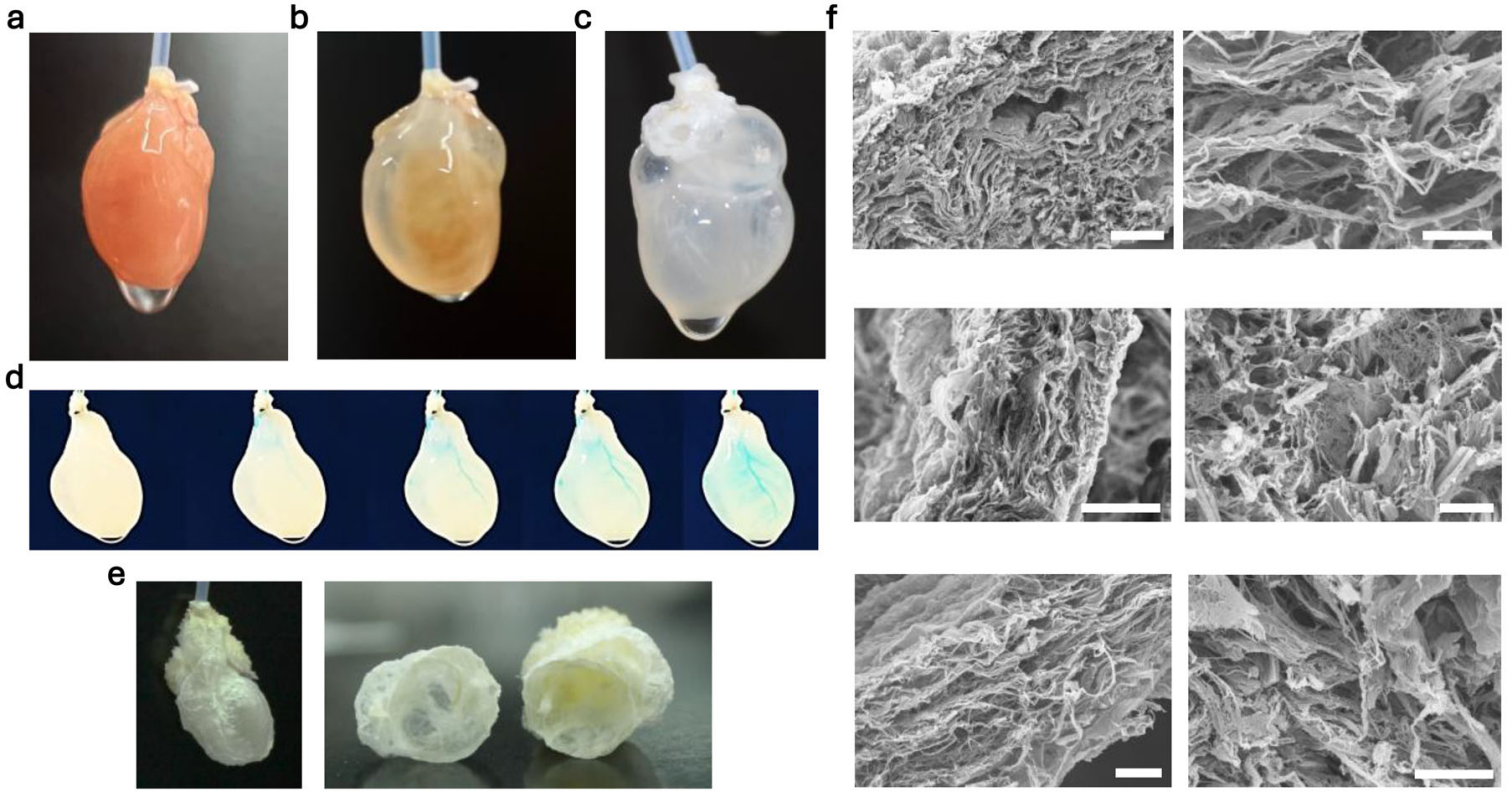
Decellularization of the native heart and preservation of whole‐heart scaffold architecture. (a–c) Representative images of the native rat heart throughout the decellularization process: native heart before decellularization (a), partially decellularized heart (b), and the final translucent acellular whole‐heart scaffold (c). (d) Perfusion of the decellularized heart with blue dye, demonstrating preservation of the coronary vascular architecture. (e) Macroscopic images of the lyophilized and sectioned decellularized scaffold, showing maintenance of the overall heart geometry and chamber organization. (f) Scanning electron microscopy images of the decellularized left ventricle (upper pictures), ventricular septum (middle pictures), and right ventricle (lower pictures), demonstrating preservation of the native extracellular matrix microarchitecture. Scale bars in (f) upper‐left and lower‐left panels, 50 µm; middle‐left panel, 100 µm; right panels, 20 µm.

### 1.2 Design of a Flexible Freestanding Electronic Device for Engineered Hearts

To integrate bioelectronics into the engineered organ, we designed a flexible, freestanding electronic device specifically tailored to the anatomical and mechanical features of the heart. A three‐dimensional digital heart model was scaled to dimensions representative of the rat heart and used as a general geometrical template. The model was then computationally unwrapped into a two‐dimensional surface projection, which served as the basis for electrode positioning and overall device architecture (Figure 3a–e). This design strategy enabled the fabrication of a heart‐conformal electronic mesh that envelops the scaffold while maintaining the porosity, flexibility, and stretchability required to accommodate cyclic cardiac motion without mechanically constraining tissue contraction.

**FIGURE 3:**
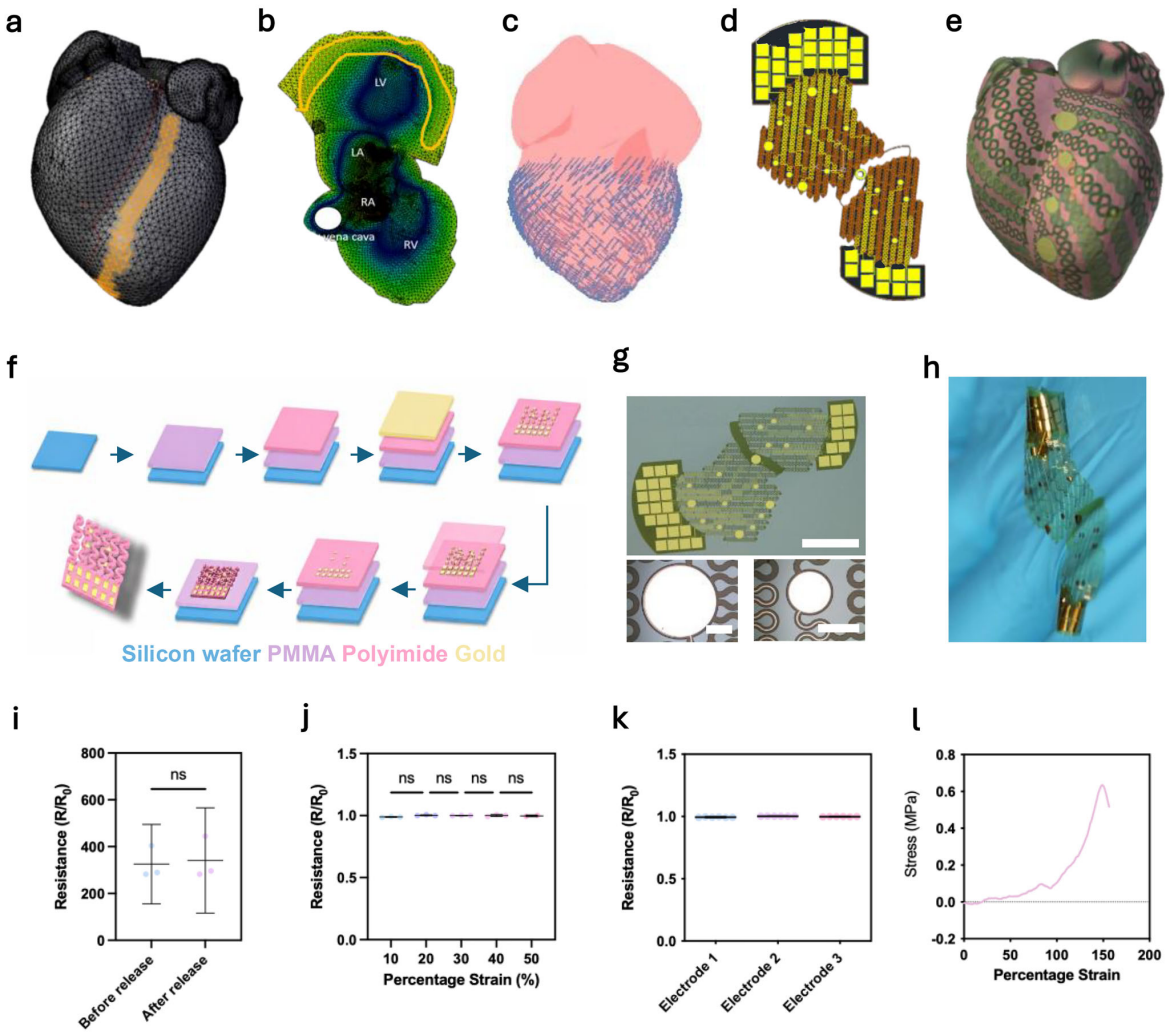
Design, fabrication, and characterization of the heart‐conformal electronic device. (a) Three‐dimensional heart model used as the anatomical basis for device design. (b) UV unwrapping of the heart model to generate a two‐dimensional representation of the cardiac surface. (c) Three‐dimensional heart model overlaid with the computationally generated contraction‐related orientation field, used to guide the general mechanical orientation of the electronic device. (d) Final layout of the heart‐conformal electronic mesh, showing electrode distribution and serpentine interconnects. (e) Computational visualization of the designed electronic mesh conformally fitted to the heart surface. (f) Schematic illustration of the microfabrication process used to generate the freestanding electronic device. (g) Representative images of the fabricated electronic mesh with magnified views of selected structural features. (h) Released freestanding electronic device. (i) Electrical resistance before and after device release from the wafer. (j) Electrical resistance as a function of applied strain. (k) Electrical resistance after 1000 deformation cycles. (l) Representative stress‐strain behavior of the freestanding device under tensile loading. Scale bars: (g) upper image, 180 µm; lower‐left image, 1 mm; lower‐right image, 550 µm.

The device layout was informed by a computationally generated contraction‐related orientation field, which guided the overall orientation of the electronic architecture (Figure 3c and Figure S3). The electrode layout was designed based on cardiac anatomy and the intended functions of the different electrode sites. Recording electrodes were distributed across spatially separated regions of the device to enable extracellular sensing from different regions of the engineered heart, whereas larger stimulation electrodes were positioned along the longitudinal extent of the heart, with particular emphasis on the interventricular septal region and regions corresponding to the sinoatrial and atrioventricular conduction pathway. Because electrode placement followed cardiac anatomy rather than a regular lattice, nearest‐neighbor center‐to‐center distances were nonuniform, with a median of 2.88 mm and a range of 2.19–4.89 mm (Figure S5). The serpentine interconnects were incorporated to dissipate mechanical stress, tolerate repeated strain during beating, and minimize the risk of structural or electrical failure over time (Figure 3d,e). In addition, insulating polyimide layers were integrated into the device architecture to spatially confine electrical interfacing to the exposed electrode sites, thereby improving signal localization and stimulation specificity.

The electronic platform was fabricated using multilayer microfabrication and photolithography techniques and released as an ultrathin, freestanding mesh suitable for integration into the hybrid whole‐heart construct (Figure 3f–h). Electrical and mechanical characterization demonstrated stable device performance following release (Figure 3i), under increasing tensile strain (Figure 3j), and after repeated cycles of mechanical deformation (Figure 3k), confirming the robustness and compliance of the system under conditions relevant to dynamic cardiac tissues (Figure 3i). Collectively, these results establish a mechanically adaptive and electrically stable bioelectronic interface designed for seamless integration with engineered whole‐heart scaffolds.

### 1.3 Assembly of a Hybrid Bioelectronic Heart

To generate the outer hybrid component of the bioelectronic heart construct, the same heart‐derived two‐dimensional anatomical map (Figure 4a,b) used for device design was employed to fabricate a matching external layer. This layer was produced by three‐dimensional printing of a thermoresponsive ECM‐based hydrogel [3, 20, 21] in a heart‐conformal geometry designed to closely match the external contours of the native organ (Figure 4c and Figure S4). Following printing, the freestanding electronic mesh was carefully aligned and integrated onto the hydrogel layer, generating a two‐component bioelectronic intermediate (Figure 4d). Incubation at physiological temperature induced hydrogel crosslinking and promoted stable adhesion between the biomaterial and the electronic device. This assembly strategy yielded a flexible hybrid hydrogel‐electronics coating layer capable of enveloping the decellularized heart scaffold while positioning the electronics in an intramural‐like configuration within the final construct wall. By integrating soft bioelectronics into a biologically derived coating layer, this approach enabled the creation of a structurally compliant interface that maintains intimate contact with the engineered tissue while preserving mechanical adaptability during contraction.

**FIGURE 4:**
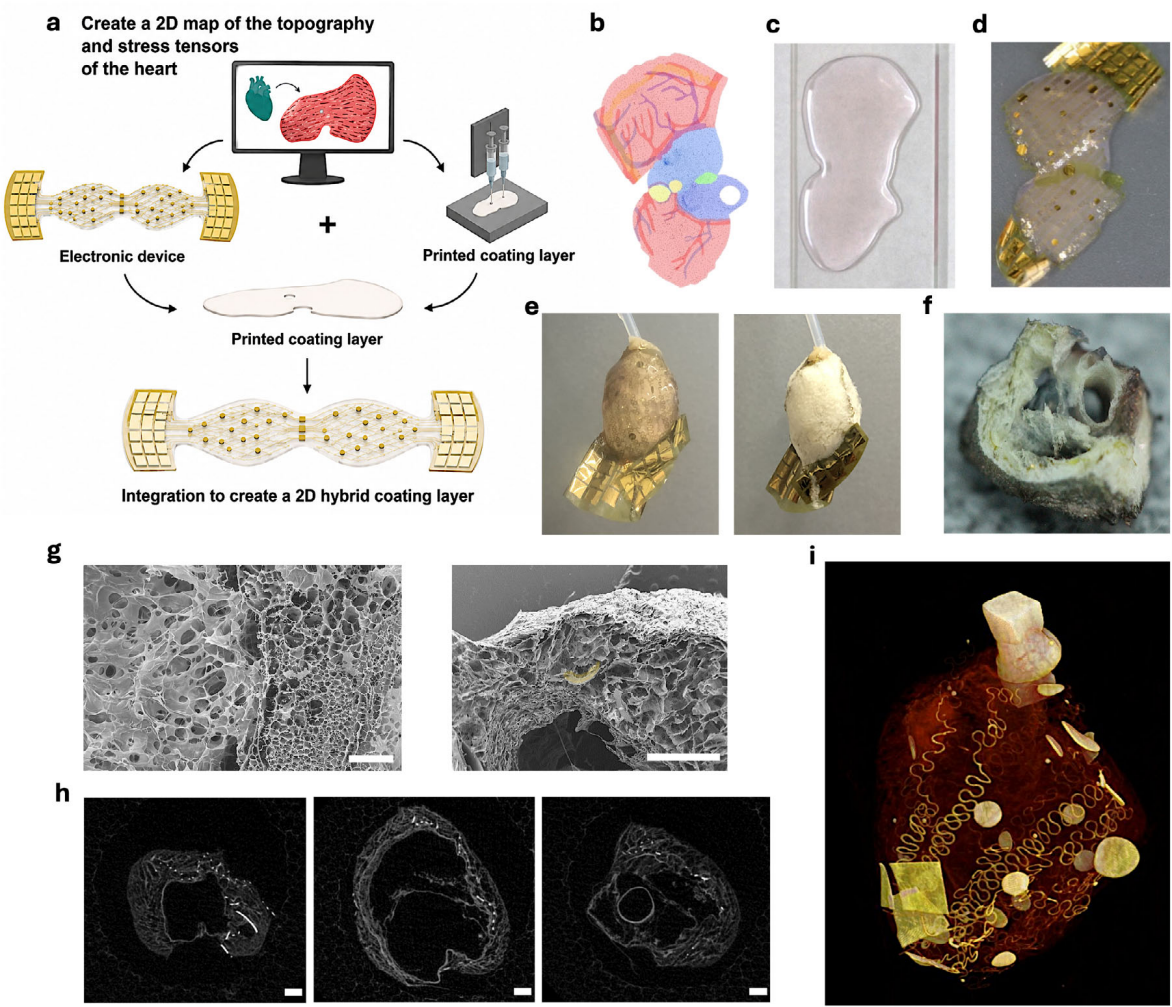
Fabrication of the hybrid hydrogel‐electronics coating layer and assembly of the whole‐heart bioelectronic scaffold. (a) Schematic illustration of the fabrication strategy used to generate the two‐dimensional hybrid coating layer by integrating the electronic device with the printed hydrogel layer. (b) Heart‐derived two‐dimensional map used to guide the design and fabrication of the coating layer. (c) Printed hydrogel coating layer. (d) Hybrid hydrogel‐electronics coating layer following integration of the electronic device with the printed biomaterial layer. (e) Macroscopic views of the assembled whole‐heart construct before and after lyophilization. (f) Cross‐sectional views of the assembled scaffold showing the position of the embedded electronics within the construct wall. (g) Scanning electron microscopy images revealing the interface between the decellularized heart scaffold, the hydrogel coating layer, and the embedded electronics. (h) Micro‐computed tomography slices of the assembled construct along the apical‐to‐basal axis, progressing from the apex on the left toward the upper/base region on the right. (i) Three‐dimensional reconstruction of the assembled whole‐heart bioelectronic scaffold. Scale bars: (g) left panel, 100 µm; middle panel, 1 mm. (h) 1 mm.

Next, the decellularized heart scaffold, the hybrid hydrogel‐electronics coating layer, and the embedded electronic mesh were assembled into a whole‐heart bioelectronic construct. In the initial stage, the acellular platform was examined to determine whether the decellularized ECM, hydrogel coating, and electronics could be integrated into a continuous and structurally coherent scaffold. Macroscopic imaging before and after lyophilization demonstrated successful formation of an organ‐scale hybrid construct while preserving the overall geometry of the heart (Figure 4e). Cross‐sectional analysis further revealed the spatial localization of the electronic mesh within the wall of the hybrid engineered scaffold, confirming successful embedding of the device throughout the construct (Figure 4f). Scanning electron microscopy demonstrated seamless integration between the native decellularized ECM, the printed hydrogel layer, and the embedded electronic components, while preserving a continuous porous fibrous microarchitecture across the different layers of the construct (Figure 4g). Micro‐computed tomography imaging and three‐dimensional reconstruction further confirmed the formation of a continuous hybrid scaffold with the electronics distributed throughout the engineered heart wall (Figure 4h,i and Video S1). Together, these analyses demonstrate the successful fabrication of a structurally integrated whole‐heart bioelectronic scaffold that seamlessly combines organ‐scale architecture with embedded electronic components in its core.

Following structural validation of the acellular platform, cellularized bioelectronic heart constructs were generated using a multilayer seeding strategy. To this end, iPSCs expressing the pluripotency markers OCT4 and Ki67 (Figure S1a–c) were efficiently differentiated into cardiomyocytes (Figure S1d,e). Cardiomyocytes were seeded into the decellularized heart scaffold by directly injecting them into the decellularized heart wall at multiple locations. In parallel, cells were encapsulated within the ECM hydrogel to generate a living bioink, which was subsequently 3D‐printed to form the outer coating layer. For the final assembly, the layers were integrated as shown in Figure S5, thereby forming a living, bioelectronic heart‐like construct.

### 1.4 Structural and Functional Characterization of the Bioelectronic Heart

The engineered bioelectronic hearts were cultivated for 14 days. Macroscopic examination demonstrated preservation of the overall hybrid organ architecture throughout the culture period, while the embedded electrodes remained visibly integrated within the heart wall, indicating stable retention of the bioelectronic interface during tissue maturation (Figure 5a).

**FIGURE 5:**
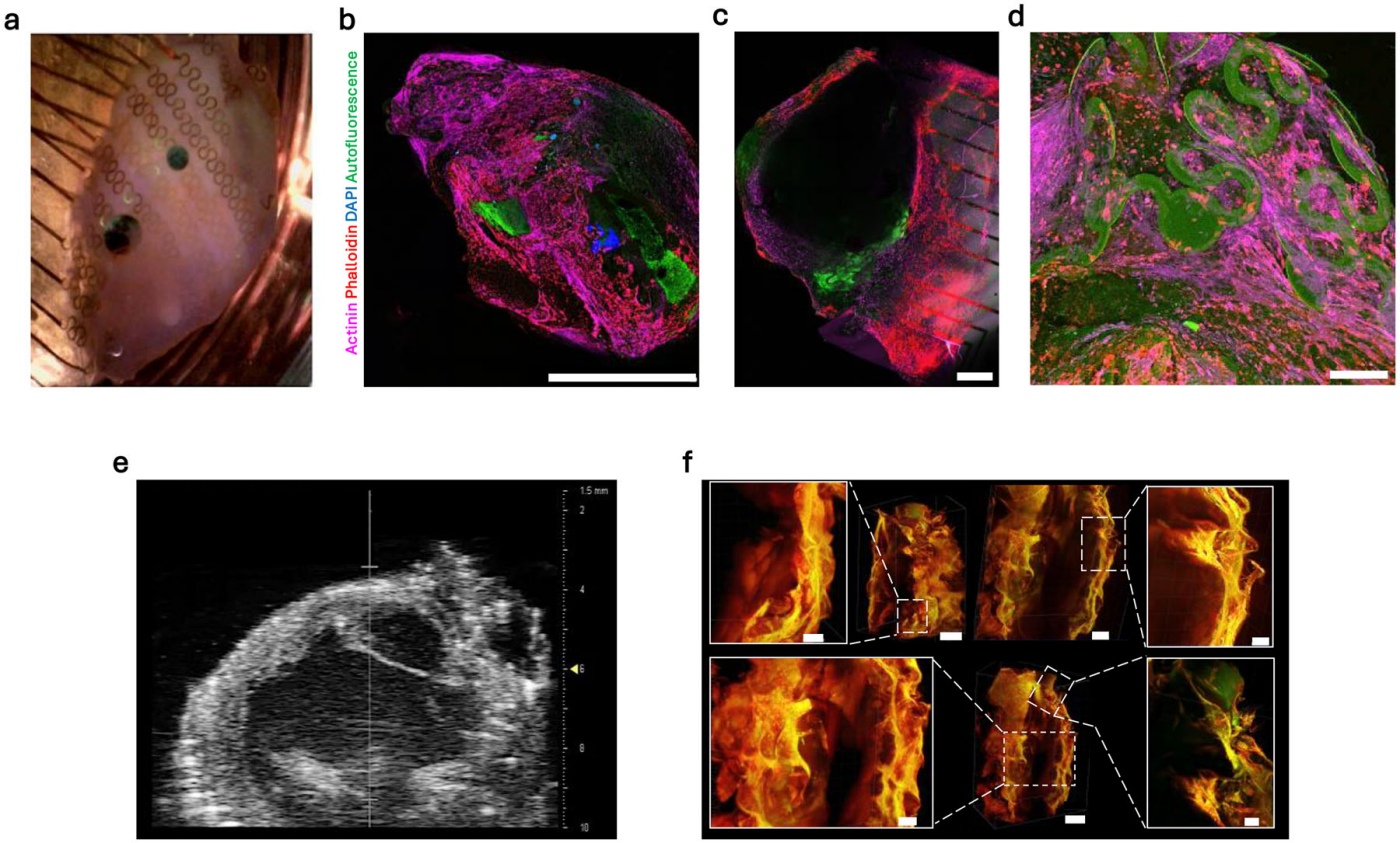
Integration between the layers of the bionic heart construct. (a) Macroscopic view of the bionic heart during culture, showing the position of the embedded electrodes on the construct. (b–d) Fluorescence imaging of the cultured construct labeled for α‐actinin, phalloidin, and DAPI, with autofluorescence from the extracellular matrix and electronic device, revealing tissue organization within the engineered heart (b), a cross‐sectional view showing the spatial organization of the engineered cardiac tissue and electronic components within the heart wall (c), and a magnified view of a representative region (d). (e) Echocardiographic image demonstrating organ‐level integration between the layers of the bionic heart construct. (f) Light‐sheet fluorescence microscopy providing three‐dimensional visualization of structural integration between the different construct layers. Scale bars: (b) 5 mm; (c) 1 mm; (d) 500 µm; (f) upper panels, 400, 1000, 600, and 250 µm from right to left; Lower panels, 400, 1000, and 250 µm from right to left.

Whole‐mount fluorescence imaging further revealed extensive cellular organization and widespread cellular distribution throughout the engineered construct (Figure 5b–d and Figure S6). Immunostaining for α‐actinin (pink), together with phalloidin labeling of the actin cytoskeleton (red), demonstrated the formation of organized cardiac tissue distributed across the hybrid scaffold and surrounding the embedded electrodes (green). Cross‐sectional imaging further demonstrated that the thickness of the engineered heart wall varied across the construct, ranging from approximately 0.4 to 1.7 mm (Figure 5c). Notably, the cells formed interconnected cardiac tissue between adjacent electrodes and exhibited pronounced sarcomeric striations, indicative of structural maturation and contractile capability. Such intimate integration of cardiac tissue with the embedded electronic network may enable efficient bidirectional communication between living cells and the bioelectronic interface.

Ultrasound imaging further demonstrated preservation of the overall hybrid heart architecture following tissue maturation, revealing a continuous and structurally coherent ventricular wall between the biological and electronic components (Figure 5e). The construct maintained a defined chamber‐like geometry with relatively homogeneous tissue organization throughout the wall thickness, suggesting stable integration of the embedded bioelectronic elements within the surrounding engineered tissue. Importantly, no major structural discontinuities or voids were observed around the electrodes within the resolution of the ultrasound images, supporting maintenance of the assembled architecture after culture.

Three‐dimensional light‐sheet fluorescence imaging further demonstrated the intimate integration of the engineered cardiac tissue with the embedded electronic architecture throughout the construct volume (Figure 5f). Volumetric reconstruction revealed dense and continuous tissue organization surrounding the electrode network, with no apparent structural separation between the biological and electronic components. High‐magnification views further showed that the tissue tightly enveloped the electrodes, supporting the formation of a stable bioelectronic interface capable of efficient electrophysiological sensing and localized electrical stimulation. Together, these complementary imaging analyses provide structural evidence of intimate integration among the different construct components, with no apparent signs of delamination after culture and several days of contractile activity.

A central objective of the bioelectronic heart concept was to endow the engineered organ with integrated capabilities for real‐time electrophysiological monitoring and active functional modulation. To evaluate this functionality, the electronic pads were connected to a multichannel amplifier system, enabling acquisition of electrical recordings from the engineered construct. Spontaneous electrophysiological activity was successfully detected at multiple recording sites across the bioelectronic heart, demonstrating that the embedded electronic network remained fully operational following tissue assembly and prolonged culture, thereby enabling organ‐level monitoring of cardiac electrical behavior (Figure 6a). Quantification of the recordings from two representative electrodes revealed mean peak‐to‐peak amplitudes of 167.0 and 31.0 µV, with corresponding signal‐to‐noise ratios of 66 and 30, respectively (Figure S7). The differences in signal amplitude between the recording sites likely reflect local differences in electrode‐tissue coupling, while the high signal‐to‐noise ratios demonstrate robust detection of cardiac electrical activity at both sites.

**FIGURE 6:**
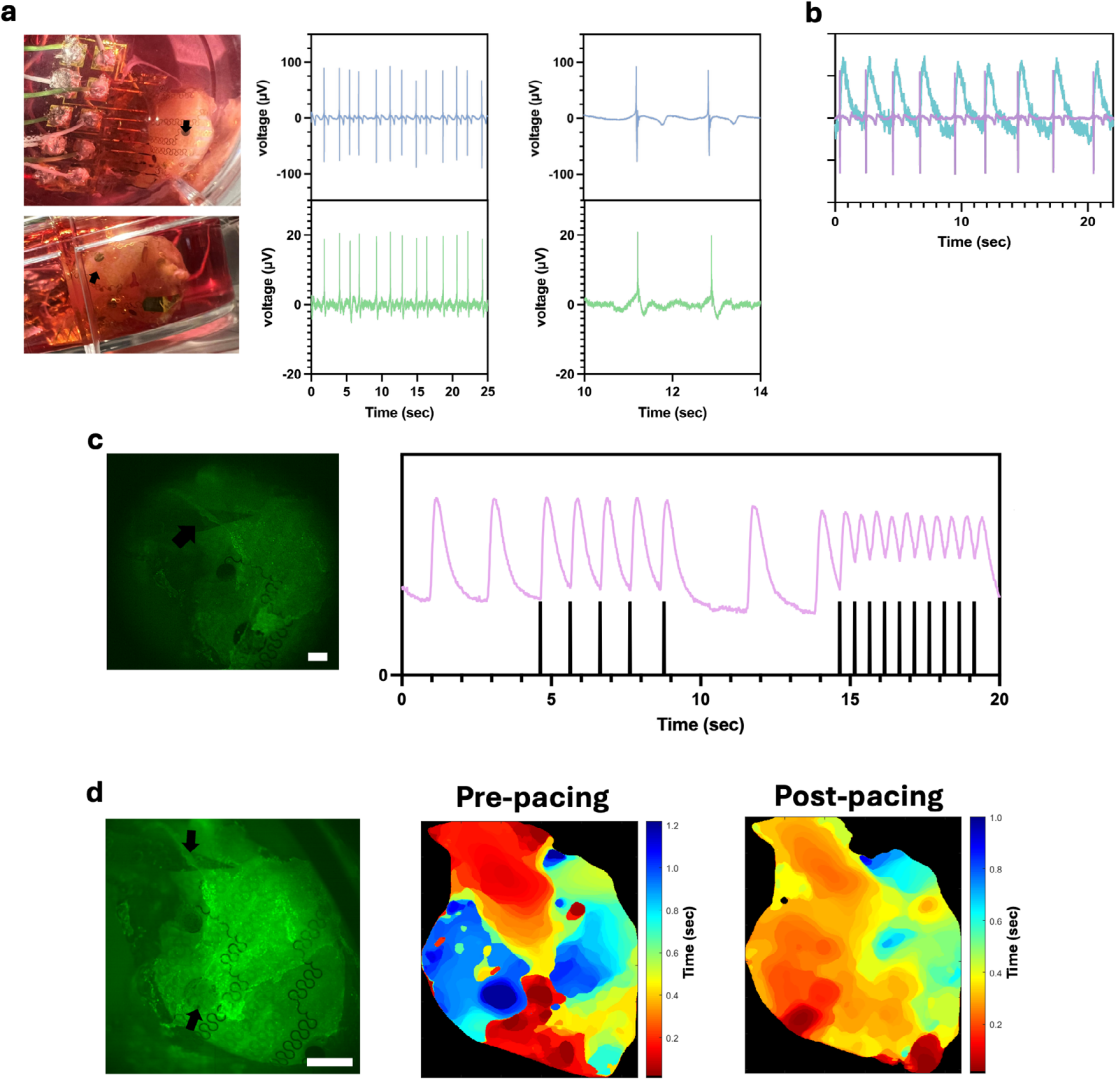
Electrophysiological monitoring and active modulation of bionic heart function. (a) Spontaneous electrical activity recorded from selected integrated electrodes within the bionic heart, shown together with the corresponding recording sites on the construct. (b) Simultaneous comparison between electrode‐based electrical recordings (purple) and calcium transients (cyan), confirming that the acquired electrical signals reflect intrinsic cardiac activity of the engineered tissue. (c) Local point stimulation evokes a corresponding calcium response in the bionic heart, shown as calcium transients (magenta) together with the applied stimulation pulses (black; 1 and 2 Hz). The arrow indicates the stimulation site. (d) Calcium‐based activation heat maps recorded before and after electrical pacing, demonstrating altered calcium‐wave propagation patterns following stimulation. Arrows indicate the stimulation sites used for pacing. Scale bars: (c) 1 mm; (d) 2 mm.

To verify that the recorded electrical signals originated from intrinsic biological cardiac activity, extracellular electrophysiological recordings were correlated with calcium transients acquired from the same spontaneously beating construct (Figure 6b). Simultaneous optical and electrical measurements demonstrated strong temporal synchronization between calcium cycling dynamics and the recorded extracellular field potentials, confirming that the embedded electrodes faithfully captured the spontaneous electrophysiological activity of the engineered cardiac tissue. These findings further establish robust functional coupling between the living cardiac network and the integrated bioelectronic interface, highlighting the platform's capability for real‐time monitoring of tissue‐level electromechanical behavior. Across three independent bioelectronic heart constructs, spontaneous calcium transients exhibited a mean frequency of 0.65 ± 0.13 Hz. The mean full width at half maximum (FWHM) was 0.528 ± 0.094 s, and the mean decay time constant (τ) was 0.449 ± 0.039 s. Under spontaneous conditions, activation across the construct was heterogeneous and lacked a stable, coordinated pattern, with activity arising from multiple local foci.

We next investigated whether the embedded electronics could be utilized not only for passive monitoring but also for active modulation of tissue function. Localized electrical stimulation delivered via selected embedded electrodes successfully captured and paced the engineered tissue at both 1 and 2 Hz, as evidenced by synchronized calcium responses in the surrounding cardiac tissue (Figure 6c). The evoked calcium transients closely followed the timing of the applied stimulation protocol, confirming effective electrical coupling between the electrodes and the living tissue and enabling precise external control over tissue activation dynamics.

To further evaluate the impact of stimulation on organ‐level electrophysiological behavior, spatial activation mapping was performed using calcium imaging rather than the integrated electrode array (Figure 6d). The embedded electrodes were used for localized electrophysiological recording and stimulation, whereas calcium imaging provided the spatial information required to generate the activation maps. The calcium‐based propagation maps revealed substantial reorganization of excitation patterns following electrical stimulation, including changes in activation directionality, conduction pathways, and the spatial distribution of propagating signals throughout the construct. Region‐of‐interest fluorescence traces further confirmed altered activation timing and propagation dynamics between spontaneous and pacing‐driven states. Collectively, these findings demonstrate that the embedded bioelectronic system supports both continuous electrophysiological monitoring and active functional regulation of the engineered heart tissue, establishing a biohybrid platform capable of bidirectional communication with living cardiac networks.

## 2 Discussion

The development of engineered cardiac tissues with integrated sensing and regulatory capabilities is becoming increasingly important as tissue‐engineered heart constructs grow in structural and functional complexity, creating a need for platforms that can not only support tissue formation but also continuously monitor and modulate organ‐level behavior in real time. In the present study, we established a bioelectronic whole‐heart platform that integrates living cardiac tissue with an embedded electronic network capable of both electrophysiological sensing and active stimulation. Importantly, the electronics were not simply incorporated into the construct as passive elements but formed an intimate, functional interface with the engineered tissue, enabling bidirectional communication between the living cardiac network and the embedded device architecture.

Our findings demonstrate that flexible bioelectronics can be stably integrated throughout a three‐dimensional engineered organ while preserving tissue organization, structural continuity, and electromechanical functionality. The embedded electronic mesh remained intimately associated with the surrounding tissue throughout culture, while the construct maintained coherent organ‐scale architecture and widespread cardiac tissue formation. Functional experiments further demonstrated the system's ability to record spontaneous electrophysiological activity across multiple anatomical regions and to actively modulate tissue behavior via localized electrical stimulation and pacing. Together, these results establish the feasibility of combining organ‐scale cardiac tissue engineering with integrated sensing and stimulation technologies within a single hybrid platform.

The significance of this capability extends beyond simple electrophysiological monitoring. Embedded bioelectronics may ultimately provide new opportunities to dynamically guide tissue maturation, synchronize excitation patterns, correct conduction abnormalities, or modulate organ‐level behavior in engineered cardiac tissues [17, 22, 23, 24]. More broadly, integrating electronics directly into living engineered organs provides a framework for continuously monitoring and regulating tissue development and function throughout culture and maturation [16, 18].

Despite these advances, the current platform represents an early‐stage bioelectronic heart model, and several important challenges remain before clinically relevant engineered hearts can be realized. Among these is the need to move beyond the rat‐heart scale used in the present study. One potential route would be to extend the decellularized‐scaffold strategy to appropriately selected human donor hearts that are unsuitable for transplantation. Such hearts could be decellularized and subsequently repopulated with human iPSC‐derived cardiac cells [12] and integrated with bioelectronic components. Porcine hearts could provide an important intermediate large‐animal model for adapting cellularization, biofabrication, electronic design, and long‐term culture strategies that are required at larger scales. Alternatively, direct three‐dimensional bioprinting may provide another route toward the generation of whole bioelectronic hearts, in which both the living tissue and the electronic components are integrated during the bioprinting process [3, 13, 25, 26]. Importantly, this approach could also provide control over the overall dimensions and geometry of the construct, potentially enabling patient‐specific anatomical customization. Such approaches could enable even more intimate spatial integration between cardiomyocytes, extracellular matrix components, vascular structures, and embedded electronics, allowing precise positioning of sensing and stimulation elements throughout the construct volume. The convergence of advanced biofabrication and printable bioelectronics may ultimately provide unprecedented control over tissue‐electronics interfacing at the organ scale, enabling the generation of engineered hearts with highly distributed sensing, adaptive stimulation, and programmable functional regulation capabilities.

At the current stage, further development of the present platform will require improvements in cellularization, vascular integration, long‐term perfusion culture, tissue maturation, and electromechanical synchronization. These advances will be necessary to achieve greater maturation, coordinated chamber‐level contraction, and effective fluid ejection and pump function. Future constructs will also likely require greater biological complexity, including vascular, stromal, neuronal, and immune‐supportive cell populations to better reproduce the multicellular environment of the native myocardium. The heterogeneous spontaneous activation observed in the present constructs likely reflects the use of a non‐regionally patterned hiPSC‐derived cardiomyocyte population and the absence of an anatomically organized specialized conduction system. Accordingly, region‐specific organization of pacemaker, atrial, ventricular, and specialized conduction‐system cell populations within anatomically defined cardiac compartments may be important for establishing a more physiologically coordinated intrinsic activation sequence and recreating chamber‐specific electrophysiological and mechanical behavior.

The bioelectronic dimension of the platform itself can likewise be further expanded. Future systems may incorporate closed‐loop feedback architectures capable of dynamically responding to tissue state in real time, as well as multimodal sensing modalities for monitoring electrophysiological, mechanical, metabolic, or biochemical tissue behavior. In addition, future device architectures may integrate localized therapeutic delivery capabilities for spatially controlled release of bioactive molecules to promote vascularization, improve tissue maturation, or provide localized functional rescue. Such approaches suggest a future direction in which engineered organs are not only structurally fabricated, but are also continuously monitored, electronically regulated, and therapeutically modulated throughout their development and functional lifetime.

Overall, this work establishes a structural, biological, and bioelectronic framework for the generation of engineered whole‐heart constructs with integrated sensing and stimulation capabilities. While substantial advances remain necessary before achieving clinically functional bioengineered hearts, the present study provides a foundational step toward the emerging field of bioelectronic organ engineering and toward the development of engineered cardiac tissues with enhanced functionality, controllability, and therapeutic potential.

## 3 Experimental Section

Selected experimental methods are presented below. The full methodology is provided in the Supporting Information.

### 3.1 Whole‐Heart Decellularization

Hearts were extracted from dead Sprague‐Dawley rats and cannulated via the ascending aorta using a prefilled 21 G cannula to enable retrograde coronary perfusion. Hearts were first perfused with PBS containing heparin (500 U L^−^^1^) for 30 min, followed by PBS perfusion for 30 min. Decellularization was then performed by perfusion with 1% SDS (Sigma–Aldrich) in DDW for 24–48 h, while the hearts were immersed in the same solution. The hearts were then perfused with 1% Triton X‐100 in DDW (Sigma–Aldrich) for 1 h, followed by washes with PBS. All solutions were either filtered or autoclaved before use.

### 3.2 Generation of a Heart‐Derived 2D Map

A three‐dimensional heart model was downloaded from Thingiverse (“Anatomical Human Heart” by 517860 (modified), under the Creative Commons—Attribution—Share Alike license—CC BY‐SA 3.0 https://creativecommons.org/licenses/by‐sa/3.0/) and processed in Blender (Blender Foundation) to generate a two‐dimensional projection of the cardiac surface. Briefly, a septal incision was defined using the seam function, and the model was converted into a two‐dimensional heart map using UV unwrap. This map was used for the design of the electronic mesh, electrode positioning, and fabrication of the matching coating layer. To further guide the mechanical orientation of the electronic device, cardiac fiber orientation was computationally approximated using simplified ellipsoid‐based and anatomical heart‐surface models. Local surface normals, the longitudinal heart axis, and circumferential directions were used to generate a contraction‐related orientation field across the heart surface. This field was then used to inform the layout and orientation of the heart‐conformal electronic mesh, aiming to align device mechanics with the expected anisotropic contraction pattern of the myocardium.

### 3.3 Fabrication of the Freestanding Electronic Device

The electronic device was fabricated as previously described by Feiner et al. [15]. Briefly, the device was fabricated on a silicon wafer coated with a sacrificial PMMA layer. A flexible polyimide‐gold‐polyimide multilayer was produced by spin‐coating polyimide, depositing and patterning Cr/Au conductive traces, and encapsulating the metal layer with a second polyimide layer. Electrode pads and the open‐web geometry were defined by photolithography and oxygen plasma reactive ion etching, after which the devices were released by dissolving the PMMA layer in acetone.

### 3.4 Electrical and Mechanical Characterization of the Electronic Device

Mechanical characterization of the devices was performed using a model LS1 tensile testing instrument (Lloyd Instruments, Ltd.) equipped with a 5 N load cell, at a constant extension rate of 5 mm min^−^^1^. Electrical resistance was measured using a DMM‐7510 multimeter (Keithley Instruments, Cleveland, OH, USA) in a two‐probe setup.

### 3.5 Fabrication of the Hydrogel‐Electronics Coating Layer

The coating layer was fabricated by extrusion‐based 3D printing of 1% omental ECM hydrogel using a 3DDiscovery printer (regenHU, Switzerland). The hydrogel was extruded through a 25G needle onto a glass slide in 4 consecutive layers. Following printing, the freestanding electronic mesh was manually aligned onto the printed coating layer and incubated at 37°C for 30 min to enable hydrogel crosslinking and adhesion between the biomaterial and the electronics, thereby forming a hybrid coating layer.

### 3.6 Assembly of Cellularized Bionic Heart Constructs

For cellularized construct assembly, hiPSC‐derived cardiomyocytes were detached from culture plates using TrypLE Select (Thermo‐Fisher Scientific), collected as a single‐cell suspension, and allocated for three applications: seeding of the decellularized heart, printing of the cell‐laden coating layer, and formation of the fibrin‐based adhesive interface. For heart seeding, 15 × 10^6^ cells per heart were resuspended in Matrigel 5% and injected into the decellularized heart wall from multiple locations using a 1 mL syringe fitted with a 25 G low dead‐space needle. In parallel, the coating layer was printed using omental ECM hydrogel at a cell concentration of 45 × 10^6^ cells mL^−^^1^ hydrogel, corresponding to 20 × 10^6^ cells per patch. Following printing, the electronic mesh was placed on the coating layer and incubated at 37°C to allow gel stabilization. For final assembly, the seeded heart and the cell‐laden coating layer were joined using a fibrin‐based adhesive prepared from fibrinogen and thrombin (Sigma–Aldrich). The fibrinogen phase contained 33 mg mL^−^^1^ fibrinogen and 5 × 10^6^ cells mL^−^^1^. Thrombin was diluted to 10 U mL^−^^1^ and added at a 1:3 ratio relative to the fibrinogen phase, yielding a final mixture containing 3.75 × 10^6^ cells mL^−^^1^, 2.5 U mL^−^^1^ thrombin, and 24.75 mg mL^−^^1^ fibrinogen. The assembled constructs were then transferred to maturation medium [26] and cultured for 14 days.

### 3.7 Microcomputed Tomography

Microcomputed tomography of the assembled heart constructs was performed using a SkyScan 1176 micro‐CT system (Bruker micro‐CT, Kontich, Belgium) at a nominal pixel size of 36 µm, with a 45 kV X‐ray tube voltage and a 0.2 mm aluminum filter. Three‐dimensional surface‐rendered models were generated using CTVol, and image reconstruction was performed using NRecon v.1.6.9 (Bruker micro‐CT).

### 3.8 Light‐Sheet Imaging and Tissue Clearing

For whole‐mount light‐sheet imaging, constructs were prepared using an adapted iDISCO‐based immunostaining and clearing protocol [27]. Briefly, fixed samples were washed in PBS, dehydrated through a graded methanol series, and treated with dichloromethane (DCM) and hydrogen peroxide to improve optical clarity and reduce background signal. Samples were then rehydrated, permeabilized, blocked, and incubated with primary and secondary antibodies under gentle shaking. Following staining, samples were embedded in 2% low‐melting agarose, dehydrated again in methanol, cleared using DCM, and transferred to ethyl cinnamate (ECi) until becoming optically transparent. Light‐sheet imaging was performed using a Bruker MuVi SPIM light‐sheet microscope. Raw data were processed using Lux Processor software (Bruker) and analyzed using Imaris Viewer (Oxford Instruments).

### 3.9 Ultrasound Imaging

Echocardiographic assessment of the engineered constructs was performed using a Vevo 3100 imaging system (FUJIFILM VisualSonics).

### 3.10 Calcium Imaging and Signal Analysis

Samples were incubated with 10 µM Fluo‐4 AM (Invitrogen) and 0.1% Pluronic F‐127 (Sigma–Aldrich) in the culture medium for 30 min at 37°C. Imaging was performed using a SMZ18 binocular microscope (Nikon) and an ORCA‐Flash 4.0 digital CMOS camera (Hamamatsu Photonics) at 100 frames s^−^^1^. Videos were processed in ImageJ/FIJI by subtracting the median pixel intensity across the full time series. Calcium datasets were further analyzed to generate activation maps and region‐of‐interest fluorescence traces for comparison of spontaneous and stimulation‐driven activity.

### 3.11 Electrophysiological Recordings and Electrical Stimulation

Extracellular electrical signals were recorded from the embedded electrodes using a Model 1700 Differential AC Amplifier (A‐M Systems) connected to a USB X Series multifunction data acquisition device (National Instruments). Recordings were acquired using LabVIEW Express software. To assess active modulation, electrical pacing was applied through the integrated electrodes using a STG‐4002 stimulus generator (Multichannel Systems). Pacing was performed using pulses of 50 ms duration at 1–2 Hz, with stimulation amplitudes of up to 5 V. These values represent the experimental pacing conditions rather than a measured capture threshold. Signal filtering and analysis were performed using MATLAB, and graph generation was carried out using GraphPad Prism.

## Author Contributions

**Itai Cabilly**: investigation, writing – original draft, methodology. **Hadas Oved**: investigation, writing – original draft, methodology, visualization. **Ron Feiner**: conceptualization, investigation. **Nadav Noor**: investigation. **Moran Yadid**: investigation. **Melanie Flury**: investigation. **Assaf Shapira**: investigation. **Tal Dvir**: conceptualization, funding acquisition, validation, writing – review and editing, supervision, resources.

## Conflicts of Interest

The authors declare no conflicts of interest.

## Supporting information




**Supporting File 1**: advs78395‐sup‐0001‐MovieS1.mp4.

**Supporting File 2**: advs78395‐sup‐0002‐SuppMat.docx.

## Data Availability

The data that support the findings of this study are available on request from the corresponding author. The data are not publicly available due to privacy or ethical restrictions.
