# Supplementary material for: Engineering a Bioelectronic Hybrid Heart with Integrated Sensing and Electrical Modulation
Source: Adv Sci (Weinh). 2026 Oct 22:e78395. Online ahead of print. doi: 10.1002/advs.78395 (PMC13690982; doi:10.1002/advs.78395)
Supplement: Supplementary file 1 [file ADVS-9999-e78395-s002.docx]

**Engineering a Bioelectronic Hybrid Heart with Integrated Sensing and Electrical Modulation**

Itai Cabilly^1,2,3,#^, Hadas Oved^1,2,3,#^, Ron Feiner^1^, Nadav Noor^1^, Moran Yadid^1^, Melanie Flury^1^, Assaf Shapira^1,2,3^, Tal Dvir^1,2,3,4,5,^*

^1^The Biotechnology Unit, Shmunis School of Biomedicine and Cancer Research, Faculty of Life Sciences, Tel Aviv University, Tel Aviv 6997801, Israel

^2^The Center for Nanoscience and Nanotechnology, Tel Aviv University, Tel Aviv 6997801, Israel

^3^Sagol Center for Regenerative Biotechnology, Tel Aviv University, Tel Aviv 6997801, Israel

^4^Sagol School for Neuroscience, Tel Aviv University, Tel Aviv 6997801, Israel

^5^School of Biomedical Engineering, Faculty of Engineering, Tel Aviv University, Tel Aviv 6997801, Israel

*Correspondence: tdvir@tauex.tau.ac.il

**Supporting Information Methods**

**DNA Visualization and Quantification**

To evaluate decellularization efficiency, small samples of native and decellularized tissues were stained with Hoechst 33258 (Sigma–Aldrich) for 3 min, followed by three washes with PBS. Samples were visualized using an inverted fluorescence microscope (Nikon Eclipse Ti). For DNA quantification, genomic DNA was extracted from three random dry samples (25–30 mg) of native and decellularized tissues using the DNeasy Blood & Tissue Kit (Qiagen) according to the manufacturer’s instructions. DNA content was quantified by measuring the optical density at 260 nm using an Infinite M200 Pro plate reader (TECAN).

**Synthesis of the ECM-Based Hydrogel**

Porcine omenta (Kibbutz Lahav, Israel) were decellularized as previously described.[1–3] Briefly, tissues were washed with phosphate-buffered saline (PBS), large blood vessels were removed, and the tissue was incubated for 1 h in hypotonic buffer containing 10 mM Tris·HCl (Hy Laboratories), 5 mM EDTA (Sigma–Aldrich), and 1 µM phenylmethanesulfonyl fluoride (Sigma–Aldrich), pH 8.0, followed by three freeze–thaw cycles at −80 °C. Samples were then gradually dehydrated in 70% ethanol, 96% ethanol, and acetone (Bio-Lab), defatted in 40% acetone in n-hexane (Bio-Lab), and further treated with 0.25% trypsin-EDTA (Sigma–Aldrich), 1.5 M NaCl (Bio-Lab), and 50 mM Tris·HCl containing 1% Triton X-100 (Sigma–Aldrich) to remove residual cellular material and lipids. The resulting decellularized tissue was washed thoroughly with PBS and DDW, frozen, and lyophilized. The dry decellularized omentum was ground into flakes and enzymatically digested in 0.1 M HCl containing porcine pepsin (Sigma–Aldrich) at a ratio of 1:10 (w/w) relative to dry ECM for 3–4 days at room temperature under stirring. The pH was subsequently adjusted to 7.4 using 5 M NaOH, and 10× DMEM/F12 (Sigma–Aldrich) was added to obtain a final omental ECM concentration of 1% (w/v). The hydrogel was then filtered through a sterile 70 µm nylon cell strainer (Bar Naor), supplemented with 0.1% penicillin/streptomycin (Sigma), and stored at 4 °C until use.

**Design and Fabrication of the Electronic Device (detailed)**

The electronic device was designed using AutoCAD software (Autodesk) and fabricated on silicon wafers (University Wafers) using standard cleanroom microfabrication processes. Prior to fabrication, silicon wafers were sequentially cleaned with acetone, isopropanol, and deionized water, followed by dehydration on a hot plate for 1 min. The wafers were then treated with oxygen plasma for 5 min to improve surface activation. A sacrificial release layer was prepared by spin-coating poly(methyl methacrylate) (PMMA 495 A2, MicroChem) onto the silicon wafer at 3000 rpm for 40 s, followed by baking at 180 °C for 1 min. This process yielded an approximately 300 nm-thick PMMA layer. A first polyimide layer was then formed by spin-coating PI2525 precursor at 3000 rpm for 40 s, followed by thermal curing at 220–225 °C for 6–8 h. The cured polyimide layer had a thickness of approximately 5 µm.

Following formation of the base polyimide layer, metallic conductive traces were deposited by electron-beam evaporation using a VST e-beam evaporator. A 10 nm chromium adhesion layer and a 200 nm gold layer were deposited under high vacuum. Gold was evaporated from a tungsten boat at 1–3 × 10⁻⁶ Torr and a deposition rate of approximately 2.5 Å s⁻¹. The metal layer was subsequently patterned by photolithography. Briefly, AZ1518 photoresist was spin-coated at 3000 rpm for 40 s and baked at 115 °C for 90 s on a hot plate. The electrode and interconnect pattern was defined using the first photomask, followed by development in AZ 726 developer. The exposed Au and Cr layers were then sequentially removed by wet etching using gold and chromium etchants, respectively. After metal etching, the remaining photoresist was stripped with acetone, followed by rinsing with isopropanol and deionized water. For device encapsulation, the patterned wafers were dehydrated and treated again with oxygen plasma for 5 min. A second PI2525 layer was spin-coated at 3000 rpm for 40 s and thermally cured at 220–225 °C for 6–8 h. Electrode contact sites were opened using a second photolithography step. AZ4620 photoresist was spin-coated at 3000 rpm for 40 s, baked at 115 °C for 90 s, and exposed through a second photomask designed to protect the device while leaving the electrode contact regions accessible. The photoresist was developed in AZ 400K developer diluted 1:2 in deionized water. The exposed polyimide above the electrode pads was removed by oxygen plasma reactive ion etching using 200 mTorr pressure, 20 sccm O₂ flow, and 150 W RF power for approximately 40 min. The open-web geometry of the device was then defined using an additional photolithography and etching process. AZ4620 photoresist was applied as a thick protective mask by sequential spin-coating steps at 3000 rpm for 40 s, each followed by baking at 115 °C for 90 s. The third photomask was used to protect the final device geometry, while the surrounding polyimide was removed by oxygen plasma reactive ion etching under the same conditions described above for approximately 60 min. After completion of the etching process, the remaining photoresist was stripped using acetone, isopropanol, and deionized water. Finally, the devices were released from the silicon wafer by dissolving the underlying PMMA sacrificial layer in acetone for approximately 20–30 min. Following release, the free-standing devices were gently collected from the wafer surface and dried.

**Computational Modeling of Cardiac Fiber Orientation for Heart-Conformal Device Design**

To guide the geometry and orientation of the heart-conformal electronic device, a computational model was used to approximate the native orientation of epicardial fibers. The model was based on a local surface-coordinate approach adapted from Poisson-interpolation-based methods previously used for cardiac fiber-orientation mapping.[4] Briefly, simplified ventricular geometries were first generated as truncated ellipsoids, including a thick-walled single-ventricle model and a bi-ventricular truncated ellipsoid model. Each surface was discretized into local patches, and for each patch, a local coordinate system was defined using the longitudinal axis of the heart, the surface normal, and the corresponding circumferential direction.

For each patch, the circumferential vector was calculated as the cross product between the longitudinal axis and the local surface normal. A prescribed fiber-orientation angle was then defined relative to this circumferential direction in the circumferential longitudinal plane, consistent with the known helical arrangement and transmural variation of myocardial fiber orientation. The resulting projected fiber vector was subsequently transformed onto the local tangent plane of the heart surface, ensuring that the calculated fiber-orientation vectors followed the curved geometry of the ventricular wall. For the bi-ventricular model, the same calculation was applied to both ventricular-like domains to generate a continuous contraction-related orientation field.

An anatomical heart geometry was then used to map the resulting orientation field onto a more realistic heart surface. The surface mesh was represented by vertices and triangular faces, from which local surface normals were calculated using cross products between normalized edge vectors. After defining the longitudinal axis of the heart, the same local coordinate-based calculation was applied to each surface element. The generated vector field was used to guide the orientation and mechanical layout of the electronic mesh, enabling the device to conform to the expected contraction-related architecture of the heart.

**iPSC Maintenance and hiPSC-CM Differentiation**

Human induced pluripotent stem cells (iPSCs), originally derived from omental stromal cells, were maintained on Matrigel-coated culture dishes (BD) in NutriStem medium (Sartorius) supplemented with 0.1% penicillin/streptomycin at 37 °C in a humidified atmosphere containing 5% CO₂. Medium was replaced daily, and cells were passaged at approximately 80% confluence using ReLeSR (StemCell Technologies).

For cardiac differentiation, iPSCs were dissociated with Accutase (StemCell Technologies) and seeded onto Matrigel-coated 6-well plates. Differentiation was initiated 2 days after seeding. On day 0, cells were cultured in RPMI (Sartorius) supplemented with B27 minus insulin (Invitrogen), 1mM L-glutamine, and 4.5 µM CHIR-99021 (Tocris). On day 2, the medium was replaced with RPMI/B27 minus insulin containing 1mM L-glutamine and 5 µM IWP-2 (Tocris). On days 4 and 6, cells were maintained in RPMI/B27 minus insulin supplemented with 1mM L-glutamine, followed by RPMI/B27 supplemented with 1mM L-glutamine on days 8 and 10. Cells were collected for experiments on day 12.

**Immunostaining and Fluorescence Imaging**

Differentiated cells and engineered constructs were fixed in 3.6% formaldehyde, permeabilized with 0.1% Triton X-100 (Sigma–Aldrich), and blocked with PBS containing 1% bovine serum albumin and 10% fetal bovine serum. Samples were then incubated with the relevant primary antibodies followed by the corresponding secondary antibodies. Antibodies used for fluorescent staining included anti-α-sarcomeric actinin (Abcam), anti-connexin 43 (Invitrogen), anti-OCT4 (Abcam), and anti-Ki67 (Abcam). Secondary antibodies included Alexa Fluor 555- or Alexa Fluor 647-conjugated goat anti-mouse antibodies (Abcam / Jackson ImmunoResearch) and Alexa Fluor 488-conjugated goat anti-rabbit antibody (Jackson ImmunoResearch), as appropriate. F-actin was stained using phalloidin-iFluor 647 (Abcam), and nuclei were counterstained with Hoechst 33258 (Sigma–Aldrich). Samples were visualized using a Nikon Eclipse NI-E confocal microscope, and images were processed using NIS Elements software and ImageJ/FIJI.

**Flow Cytometry**

Flow cytometry analysis was performed using the Fixation/Permeabilization Staining Buffer Set (eBioscience) according to the manufacturer’s protocol. Cells were stained with antibodies against TRA-1-60, OCT4, and cardiac troponin T (cTnT) (Miltenyi Biotec). Data were acquired using a CytoFLEX flow cytometer (Beckman Coulter) and analyzed using CytExpert software.

**Assembly of the Acellular Whole-Heart Scaffold**

For generation of the acellular hybrid scaffold, the decellularized heart was first coated with a thin layer of omental ECM hydrogel, after which the hydrogel–electronics coating layer was positioned on the heart through the cannula so as to cover the scaffold. The assembled construct was incubated at 37 °C for 45 min to complete crosslinking, then frozen at −80 °C and lyophilized. This approach was used to generate continuous acellular constructs for structural characterization, including macroscopic imaging, sectioning, SEM, and micro-computed tomography.

**Electrophysiological and Calcium Imaging Data Analysis**

Electrophysiological recordings and calcium imaging datasets were processed using custom MATLAB scripts. Electrophysiological recordings acquired from the embedded electrodes were exported from LabVIEW as text files and imported into MATLAB. A time vector was generated according to the acquisition time step, and selected recording channels and time windows were inspected in the time domain. Voltage traces were analyzed in the frequency domain using fast Fourier transform to evaluate dominant signal and noise components. Baseline drift was reduced using zero-phase high-pass filtering with a fourth-order IIR filter. Several cutoff frequencies were compared, and the final filtering parameters were selected empirically to preserve cardiac-like extracellular field-potential morphology while minimizing low-frequency drift. Processed traces were further smoothed using moving-average and Savitzky–Golay filtering. Raw, intermediate-filtered, and final processed traces were compared throughout the analysis, and processed datasets were exported for plotting and downstream analysis.

Calcium imaging videos were imported into MATLAB from ND2 or AVI files and converted into grayscale image stacks. Regions of interest were manually selected on the first frame, with a fixed ROI radius used across all analyzed regions within each video. For each video, an additional ROI of the same size was selected outside the tissue and plotted as a background/control region. Pixel intensities were extracted from each ROI across all frames, and mean fluorescence intensity was calculated over time. ROI traces were processed using spatial smoothing and temporal filtering, and raw and filtered traces were compared to verify preservation of calcium transient dynamics. Baseline fluorescence was calculated from the initial recording period, and calcium traces were normalized and expressed as ΔF/F₀. Offset traces were generated for visualization of calcium dynamics across multiple regions.

Activation maps were generated from calcium imaging videos by calculating frame-to-frame fluorescence differences for each pixel. The first frame in which the fluorescence change exceeded a defined activation threshold was assigned as the local activation time. Pixels that did not exceed the threshold were displayed as non-activated/background regions. The resulting activation-time matrices were smoothed using median filtering and displayed using a fixed color scale. Activation times were converted from frame number to seconds according to the imaging frame rate. This analysis was used to compare spontaneous and stimulation-driven activation patterns within the engineered constructs.

[1]	Noor, N. *et al.* (2019). 3D Printing of Personalized Thick and Perfusable Cardiac Patches and Hearts. *Advanced Science*. https://doi.org/10.1002/advs.201900344.

[2]	Edri, R. *et al.* (2019). Personalized Hydrogels for Engineering Diverse Fully Autologous Tissue Implants. *Advanced Materials*. https://doi.org/10.1002/ADMA.201803895.

[3]	Shevach, M. *et al.* (2014). Fabrication of omentum-based matrix for engineering vascularized cardiac tissues. *Biofabrication*. https://doi.org/10.1088/1758-5082/6/2/024101.

[4]	Wong, J. and Kuhl, E. (2014). Generating fibre orientation maps in human heart models using Poisson interpolation. *Computer methods in biomechanics and biomedical engineering*. https://doi.org/10.1080/10255842.2012.739167.

**Supporting Information Figures**

**Figure S1. Characterization of hiPSCs and hiPSC-derived cardiomyocytes. a,** Representative immunofluorescence images of human induced pluripotent stem cells (hiPSCs) stained for OCT4, KI67, and Hoechst. **b,c,** Flow cytometry analysis of hiPSCs for the pluripotency markers TRA-1-60 (**b**) and OCT4 (**c**). **d,** Representative immunofluorescence image of hiPSC-derived cardiomyocytes following differentiation, stained for sarcomeric α-actinin and Hoechst. **e,** Flow cytometry analysis of differentiated hiPSC-derived cardiomyocytes for cardiac troponin T (cTnT). Scale bars: **a**, 50 µm; **d**, 1 mm.

**Figure S2. Validation of decellularization efficiency and structural preservation of the whole-heart scaffold.** **a,** Macroscopic image of the decellularized whole-heart scaffold. **b,** Cross-sectional view of the decellularized heart, showing preservation of the overall chamber geometry. **c,** Scanning electron microscopy images of the decellularized scaffold, showing preserved extracellular matrix architecture in the atrium (**left**), left ventricular wall (**middle**), and aorta (**right**). **d,** Hoechst staining of native and decellularized heart tissue, confirming efficient removal of cellular nuclei following decellularization. **e,** Quantification of DNA content in native and decellularized heart tissue, demonstrating a significant reduction in residual DNA after decellularization. Scale bars: **c**, left panel, 100 µm; middle panel, 200 µm; right panel, 500 µm; **d**, 100 µm.

**Figure S3. Computational modeling of cardiac fiber orientation for heart-conformal device design. a,** The heart was modeled as a thick-walled truncated ellipsoid, representing a simplified ventricular geometry. **b,** The heart was modeled as a bi-ventricular truncated ellipsoid to better approximate whole-heart geometry. **c,** Posterior and apical views of the contraction-related tensor field mapped onto the heart model, used to guide the orientation and mechanical design of the heart-conformal electronic device.

**Figure S4. 3D printing strategy for fabrication of the hydrogel coating layer. a,** Representative printing path used to generate each layer of the hydrogel coating layer. **b,c,** Representative images acquired during extrusion-based 3D printing of the hydrogel coating layer.

**Figure S5. Assembly strategy for the cellularized bionic heart construct. a,** Application of fibrin onto the printed hydrogel layer to improve structural stability and prevent collapse of the coating layer during assembly. **b,** Positioning of the seeded decellularized heart scaffold onto the hybrid hydrogel electronics layer. **c,** Addition of fibrinogen and thrombin followed by wrapping of the seeded heart scaffold with the hybrid coating layer. **d,** Representative macroscopic view of the assembled bionic heart construct. **e,** Layout of the second-generation electronic device used for integration within the bionic heart construct. **f,** Spatial distribution of the electrode sites within the electronic device, showing the nearest-neighbor center-to-center distances derived from the original CAD geometry. Scale bar: **e-f,** 5 mm.

**Figure S6. Immunostaining of cardiac tissue within the bionic heart construct. a–c,** Immunofluorescence images of cardiac tissue formed on the integrated electronic device, showing sarcomeric α-actinin, connexin 43, and DAPI staining. **d,e,** Immunofluorescence images of cross-sections from the bionic heart construct, demonstrating cardiac tissue organization within the scaffold. Scale bars: **a**, 100 µm; **b,c**, 20 µm; **d,e**, 50 µm.

**Figure S7. Signal processing and quantitative characterization of electrophysiological recordings acquired from integrated electrodes. a,c,** Representative raw electrophysiological recordings acquired from two integrated electrodes within the bionic heart construct, with the corresponding filtered signals overlaid. **b,d,** Magnified views of representative field-potential-like events from the corresponding recordings following signal filtering. **e,** Quantification of the peak-to-peak amplitude for the two representative electrodes. **f,** Quantification of the signal-to-noise ratio (SNR) for the same electrodes.
